# Omicron Infection Evokes Cross-Protection against SARS-CoV-2 Variants in Vaccinees

**DOI:** 10.3390/vaccines10050808

**Published:** 2022-05-19

**Authors:** Gabriele Anichini, Chiara Terrosi, Claudia Gandolfo, Gianni Gori Savellini, Simonetta Fabrizi, Giovanni Battista Miceli, Federico Franchi, Maria Grazia Cusi

**Affiliations:** 1 Virology Unit, Department of Medical Biotechnologies, University of Siena, 53100 Siena, Italy; gabriele.anichini@student.unisi.it (G.A.); chiara.terrosi@unisi.it (C.T.); gianni.gori@unisi.it (G.G.S.); 2 Virology Unit, Santa Maria alle Scotte University Hospital, V.le Bracci 1, 53100 Siena, Italy; claudia.gandolfo@unisi.it; 3 Preventive Medicine and Health Surveillance Unit, Santa Maria alle Scotte University Hospital, V.le Bracci 1, 53100 Siena, Italy; s.fabrizi@ao-siena.toscana.it (S.F.); giovanni.miceli@ao-siena.toscana.it (G.B.M.); 4 Emergency-Anesthesia and Intensive Care Unit, Department of Medicine, Surgery and Neurosciences, Santa Maria alle Scotte University Hospital, V.le Bracci 1, 53100 Siena, Italy; federico.franchi@unisi.it

**Keywords:** SARS-CoV-2, Omicron, mRNA vaccine, BNT162b2, mRNA-1273

## Abstract

Due to the rapid global spread of the Omicron (B.1.1.529) variant, efforts to scale up COVID-19 booster vaccination have been improved, especially in light of the increasing evidence of reduced neutralizing antibody (NT Ab) over time in vaccinated subjects. In this study, neutralizing antibody responses against the Wild-Type, Delta, and Omicron strains were evaluated among vaccinees, both infected with Omicron or uninfected, and non-vaccinated subjects infected with Omicron. The aim of the study was to compare the cross-protective humoral response to the variant strains induced by vaccination and/or Omicron infection. The results showed a significant difference in the neutralizing antibody response between the vaccinees and the Omicron-infected vaccinated subjects against the three tested strains (*p* < 0.001), confirming the booster effect of the Omicron infection in the vaccinees. By contrast, Omicron infection only did not enhance the antibody response to the other variants, indicating a lack of cross-protection. These results suggest the importance of updating the current formulation of the SARS-CoV-2 vaccine to protect people against the Omicron subvariants. A specific Omicron vaccine, administered as a booster for the previously adopted mRNA vaccines, may protect against a wider range of SARS-CoV-2 variants. However, it is unlikely that the Omicron vaccine alone would be able to protect non-vaccinated subjects against other circulating variants.

## 1. Introduction

To limit the spread of the severe acute respiratory syndrome–coronavirus 2 (SARS-CoV-2) and to counteract the waning of protection among vaccinated individuals [1,2,3,4,5], on 12 August 2021, the FDA authorized the use of a third booster dose with either BNT162b2 or mRNA-1273 vaccines in immunocompromised individuals [6]. Lately, due to the emergency arising from the rapid global spread of the immune-evasive Omicron (B.1.1.529) variant [7,8], first detected in South Africa and Botswana, efforts to scale up COVID-19 booster vaccination have been improved, especially after the increasing evidence of reduced neutralizing antibody (NT Ab) responses to the Omicron variant compared with the original strain of SARS-CoV-2 and the Delta (B.1.617.2) variant in vaccinees [9,10,11,12].

In this study, the neutralizing antibody response against Wild-Type, Delta, and Omicron strains was evaluated among vaccinees, both infected with Omicron or uninfected, and non-vaccinated subjects infected with Omicron. The aim of the study was to compare the cross-protective humoral responses to different SARS-CoV-2 variants induced by vaccination and/or Omicron infection.

## 2. Materials and Methods

### 2.1. Study Design and Participants

In this observational cohort study, 95 subjects (29 males, 66 females; mean age 46.0 years, range 20–68) were enrolled. Seventy-five of them (21 males, 54 females; mean age 47.2 years, range 25–63) were healthcare workers from ‘Santa Maria alle Scotte’ University Hospital in Siena, who had received a third dose of mRNA-1273 (Moderna) vaccine after a two-dose cycle of BNT162b2 vaccine (Pfizer Inc., New York, NY, USA), three months before testing (average time: 93 days, range 88–95). Among them, 25 subjects (9 males, 16 females; mean age 46.7 years, range 26–61) had a past COVID-19 diagnosis, confirmed both by PCR and serological testing (between December 2021 and January 2022, approximatively 45 days after the third dose of vaccine, range 43–48).

The remaining 20 subjects, who had never been vaccinated (8 males and 12 females; mean age 51.7 years, range 20–68; average time before the screening 45 days, range 43–48), were infected by Omicron BA.1 strain, as revealed by specific sequencing. All subjects were from the Siena area. Those infected by SARS-CoV-2 showed mild or moderate symptoms.

In order to evaluate the humoral response induced by the vaccine, a blood sample was drawn from all the subjects to detect specific IgG against SARS-CoV-2 spike receptor-binding domain (RBD). Moreover, all the selected subjects’ sera were tested for the presence of specific neutralizing antibodies against the virus variants.

Finally, neutralizing antibody titers of non-vaccinated subjects infected with the Omicron BA.1 strain were compared with those observed in 20 subjects infected by the Wuhan strain, whose sera were collected between May and August 2020 (45 days after infection, similar age and sex distribution). 

For simplicity, the date of the first SARS-CoV-2-positive swab was assumed to be the first day of infection. This research was carried out according to the principles of Helsinki declaration, with reference to BIOBANK-MIU-2010 document approved by the Ethics Committee with amendment No. 1, on 17 February 2020. Prior to participating in this study, all subjects signed a written informed consent form. 

### 2.2. SARS-CoV-2 IgG Antibody Detection

Subjects’ sera were analyzed using Abbott SARS-CoV-2 IgG II Quant assay (Abbott Laboratories, Chicago, IL, USA), a chemiluminescent microparticle immunoassay (CMIA) used as an aid in evaluating the immune status of individuals with quantitative measurement of IgG antibodies against the spike receptor-binding domain (RBD) of SARS-CoV-2. This assay was performed on an Abbott Alinity (Abbott Diagnostics) according to the manufacturer’s instructions. A sample was considered positive when the result was >50.0 AU/mL. Values higher than 40,000 AU/mL were not investigated further and reported as 40,000, as it was the limit of the kit detection. 

SARS-CoV-2 natural infection was confirmed using Abbott SARS-CoV-2 anti-nucleocapsid IgM and IgG assay (CMIA), according to the manufacturer’s instructions. The final interpretation of positivity was determined by a ratio above a threshold value, ≥1.4 relative light units (RLU).

### 2.3. SARS-CoV-2 Microneutralization Test

SARS-CoV-2 virus neutralization assay was carried out on Vero E6 cells in a 96-well microplate. Twenty-five microliters of two-fold serial dilutions (1:8 to 1:1024) of sera samples were added to an equal volume of SARS-CoV-2 Wild-Type (WT) (SARS-CoV-2/human/ITA/Siena-1/2020; GenBank: MT531537.2), Delta (B.1.617.2; SARS-CoV-2/human/ITA/TUS-Siena-40/2021; GenBank: OM736177.1), and Omicron (BA.1; SARS-CoV-2/human/ITA/TUS-Siena5324294/2022; GenBank: OM956353.1) containing 100 TCID_50_ and incubated for 90 min at 37 °C. Finally, 50 μL of Vero E6 cells suspension (2 × 10^5^ cells/mL) prepared in complete DMEM were added to each well. After incubation at 37 °C, cultures were examined daily for the presence of CPE under microscope (Olympus IX51). The 50% end-point titer was calculated using the Reed–Muench method [13]. Positive and negative control sera were included in each assay [14]. Geometric mean titers (GMTs) of the neutralization assays were calculated. Values higher than 1:1024 were not investigated further. 

### 2.4. Statistical Analysis

**Figure 1:**
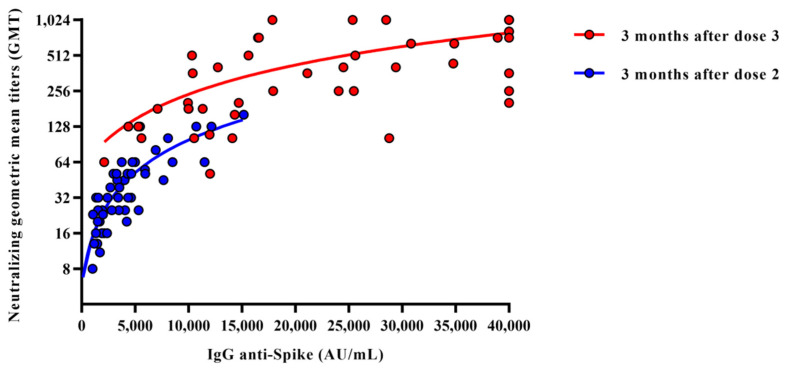
Regression analysis of the immune response in subjects three months after receiving the second or third dose of vaccine. Regression analysis of SARS-CoV-2 neutralizing antibody titers against WT, according to anti-spike IgG antibody levels (AU/mL) in serum samples obtained from subjects three months after receiving two (blue dots) or three (red dots) doses of vaccine. Regression lines for each group were calculated with slopes of 0.018 for the third-dose group and 0.009 for the second-dose group.

Differences among age, circulating IgG levels, and neutralizing geometric mean titers (GMTs) were evaluated and statistical significances were assessed with two-tailed chi-squared test. Results were considered statistically significant at *p* < 0.05. For each variable, 95% confidence interval (CI 95%) was calculated and reported. Regression analysis of neutralizing antibody IgG titers against Wild-Type strain according to the participants’ IgG anti-spike titer three months after receiving the second or third dose of vaccine was assessed (Figure 1). All analyses were performed by using Graph Pad Prism 7.0 software.

## 3. Results

Initially, the circulating anti-spike IgG and neutralizing antibody titers against the Wuhan strain (WT) were compared among the non-infected subjects three months after they received two or three doses of the vaccine (Figure 1). 

The results showed significantly higher protective titers of both circulating IgG (mean titers23,871.0 vs. 4072.0 AU/mL; CI 95% 27,563.0–20,719.0 vs. 4975.0–3178.0) and neutralizing antibody titers (GMT = 360.6 vs. 33.0, CI 95% 461.9–281.6 vs. 40.9–26.6) among those who received the third dose.

**Figure 2:**
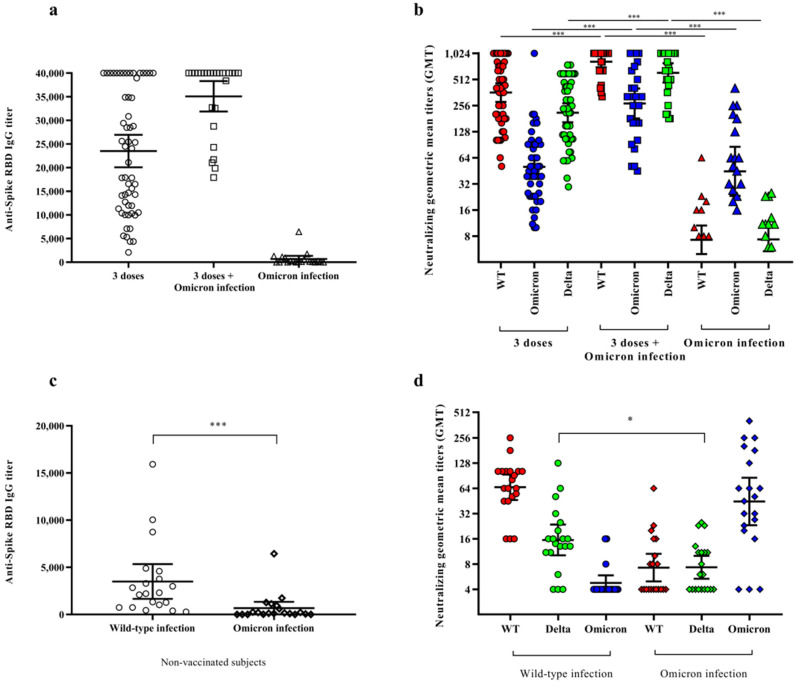
Immune response in vaccinated subjects, who either had or did not have a SARS-CoV-2 infection, and naturally infected subjects. Titers of anti-spike IgG antibodies (Panels (**a**,**c**)) and neutralizing SARS-CoV-2 antibodies (Panels (**b**,**d**)) in serum samples of subjects naturally infected with SARS-CoV-2 (triangles), vaccinated subjects with three doses of mRNA vaccine (circles), and vaccinees infected with the Omicron variant (squares). Differences in neutralizing IgG antibodies were evaluated against WT (red), Delta (B.1.617.2) (green), and Omicron (BA.1) (blue) strains (Panel (**b**)). Comparison of serum samples of WT infected subjects (circles) with those of Omicron infected subjects (rhombuses) against the three variants (Panel (**d**)). In each plot, the horizontal line represents the mean (Panels (**a**,**c**)) or the geometric mean (Panels (**b**,**d**)), while the top and bottom lines show the 95% confidence interval (CI 95%). The *p* values are reported in the figures, where * stands for *p* < 0.05 and *** stands for *p* < 0.001.

Next, in order to compare the cross-protective humoral response induced by vaccination and/or Omicron infection, we analyzed the antibody response in subjects vaccinated with three doses and subjects, vaccinated or non-vaccinated, infected with the Omicron variant. Although Omicron infection induced a low level of anti-spike RDB IgG (668.3 AU/mL, CI 95% 1346.0–0.0; Figure 2c) in the naïve subjects, it appeared to reinforce the antibody response in the vaccinated subjects, as shown by the significant increase in specific antibodies (23,519.0 vs. 35,094.0 AU/mL; CI 95% 26,966.0–20,071 vs. 38,320–31,868; *p* < 0.001) (Figure 2a). However, it is worth noting that the anti-RDB IgG levels recorded in the subjects who were naturally infected with Omicron were much lower than the level observed in those infected with WT (668.3 AU/mL vs. 3495.0 AU/mL, CI 95% 1346.0–0.0 vs. 5337.0–1563.0) (comparable mean age and time since infection; 49.3 vs. 51.7 years and 47.3 vs. 46.4 days), indicating that the common tests currently used for measuring anti-RDB antibodies do not show a good performance for all the variants (Figure 2c). Indeed, the Omicron spike contains a significant number of mutations in its RDB sequence [8], thus inducing antibodies that do not efficiently target the epitopes of the WT RDB used in this assay.

Moreover, using a live virus-based assay [13], the neutralizing antibody titers of the three groups of subjects were tested against the WT, Omicron, and Delta variants (Figure 2b). The results showed a significant difference in the neutralizing antibody response between the vaccinees and Omicron-infected vaccinated subjects against the WT (GMT = 360.6 vs. 818.6; CI 95% 461.9–281.6 vs. 951.0–704.7), Omicron (GMT = 50.2 vs. 270.2; CI 95% 65.2–38.7 vs. 402.1–181.5), and Delta strains (GMT = 211.1 vs. 607.8; CI 95% 271.3–164.3 vs. 784.1–471.1) (*p* < 0.001 for all three strains), confirming the booster effect of Omicron infection on the vaccinees, regardless of the variant tested in the neutralization assay (Figure 2b). 

By contrast, Omicron infection only, which induced a modest response in the unvaccinated subjects (GMT = 44.6, CI 95% 86.5–23.2), did not enhance the antibody response to the other variants, indicating a lack of cross-protection (GMT = 7.2 against WT, CI 95% 10.6–5.0; and 7.3 against Delta, CI 95% 10.0–5.3) in these subjects (Figure 2b). This was mainly due to the high number of mutations in the RBD domain of the spike protein, as well as in the protein itself. These results were confirmed by comparing the neutralizing response to the WT, Delta, and Omicron strains in the naturally infected people after WT (A-lineage) or Omicron (BA.1 lineage) infection. Indeed, while the antibodies of the WT-infected subjects were able to cross-react with Delta (B.1.617.2 lineage) (GMT = 15.5, CI 95% 23.6–10.1), but unable to cross-react with Omicron (GMT = 4.7, CI 95% 5.9–3.9), the antibodies of the Omicron-infected subjects did not offer protection against the WT or Delta strains. 

## 4. Discussion

The dynamics of the humoral response against SARS-CoV-2 mRNA vaccines after the second dose of SARS-CoV-2 have been widely described, especially those innon-infected subjects, either against Omicron or against other, previously circulating strains [15,16,17,18,19]. However, immunity to SARS-CoV-2 in humans is highly variable, depending on the type of vaccine, the number of doses, and the type of variant [20]; thus, it is important to understand the level of protection experienced by heterogeneous subjects during the pandemic.

In this study, we analyzed the antibody response by chemiluminescent and microneutralization assays in subjects (either infected with the Omicron BA.1 strain or not) three months after their third dose of the SARS-CoV-2 mRNA vaccine. Moreover, the humoral response was compared in naïve subjects who were infected with the Omicron or Wild-Type SARS-CoV-2 strain approximately 45 days before being screened. The cross-neutralizing antibody responses against Wild-Type, Omicron, and Delta strains were evaluated among all the groups. 

In addition, to neutralizing antibodies interfering with virion binding to receptors and blocking virus uptake into the host cells, the vaccinated subjects who were infected with Omicron developed other antibodies, causing virus particles to stick, making them easier targets for immune cells. Other antibodies could have bound to the receptors on the surfaces of the phagocytic cells, triggering the phagocytosis on the infected cells. Finally, antibodies activating the complement system, opsonizing and promoting the phagocytosis of infected cells, have been shown to play a role in viral clearance [21,22]. Therefore, we believe that a dual vaccination, based on an Omicron vaccine in addition to the current mRNA vaccine, could help to protect against SARS-CoV-2 variants, although some different amino acids are present in the spike sequence.

The results were partly confirmed by analyzing the antibody response by CMIA (Figure 2a,c). The test used for detecting the anti-RBD antibodies underestimated the presence of the anti-Omicron antibodies, probably due to the fact that they were not recognized because of the many mutations in this domain of the Omicron spike. Thus, it is necessary to update the assays based on the RBD sequence with regard to the circulating variants. 

These data confirm that the use of a potential Omicron vaccine alone would not protect against other circulating variants. Therefore, while the introduction of an Omicron-based vaccine might benefit those who have already been vaccinated with the current mRNA formulation, the same is not valid for non-vaccinated individuals, as they would not be protected against other variants.

Indeed, it is worth noting that Omicron breakthrough infection mediates a robust B-cell recall response, expanding preformed memory B cells that recognize the epitopes shared by different variants [23,24]. It has been reported that the preformed B-cell memory pool has sufficient plasticity to be remodeled by exposure to heterologous S protein, allowing the effective neutralization of other variants [25]. Therefore, we consider that breakthrough infection with Omicron could enrich the memory B-cell plateaus of vaccinated individuals and recall preexisting B cells, which recognize the conserved S protein epitopes and provide cross-protection. 

The original vaccines continue to provide neutralizing antibody responses that are relevant to the current antigenic landscape. However, the development of vaccines based on more contemporary variants may further improve the protective efficacy of vaccine-induced immune responses.

These findings support the importance of updating the current formulation of SARS-CoV-2 vaccines and provide a new vaccine against the B1.1.529 and BA lineages, in order to protect people against the Omicron subvariants, which are phylogenetically distant from the previously circulating variants. 

## Data Availability

The data supporting the reported results can be provided, upon reasonable request, by the corresponding author.
